# An oral keratinocyte life cycle model identifies novel host genome regulation by human papillomavirus 16 relevant to HPV positive head and neck cancer

**DOI:** 10.18632/oncotarget.18328

**Published:** 2017-06-01

**Authors:** Michael R. Evans, Claire D. James, Oonagh Loughran, Tara J. Nulton, Xu Wang, Molly L. Bristol, Brad Windle, Iain M. Morgan

**Affiliations:** ^1^ Department of Oral and Craniofacial Molecular Biology, VCU Philips Institute for Oral Health Research, Virginia Commonwealth University School of Dentistry, Richmond, VA, USA; ^2^ VCU Massey Cancer Center, Richmond, VA, USA

**Keywords:** head and neck cancer, human papillomavirus, life-cycle, the cancer genome atlas, oral keratinocytes

## Abstract

Many aspects of the HPV life cycle have been characterized in cervical cell lines (W12, CIN612) and in HPV immortalized primary foreskin keratinocytes. There is now an epidemic of HPV positive oropharyngeal cancers (HPV16 is responsible for 80-90% of these); therefore increased understanding of the HPV16 life cycle in oral keratinocytes is a priority. To date there have been limited reports characterizing the HPV16 life cycle in oral keratinocytes. Using TERT immortalized “normal” oral keratinocytes (NOKs) we generated clonal cell lines maintaining the HPV16 genome as an episome, NOKs+HPV16. Organotypic raft cultures demonstrated appropriate expression of differentiation markers, E1^E4 and E2 expression along with amplification of the viral genome in the upper layers of the epithelium. Using this unique system RNA-seq analysis revealed extensive gene regulation of the host genome by HPV16; many of the changes have not been observed for HPV16 before. The RNA-seq data was validated on a key set of anti-viral innate immune response genes repressed by HPV16 in NOKs+HPV16. We show that the behavior of these NOKs+HPV16 lines is identical to HPV16 immortalized human tonsil keratinocytes with regards innate gene regulation. Finally, using The Cancer Genome Atlas (TCGA) data we examined gene expression patterns from HPV positive and negative head and neck cancers and demonstrate this innate immune gene signature set is also downregulated in HPV positive cancers versus negative. Our system provides a model for understanding HPV16 transcriptional regulation of oral keratinocytes that is directly relevant to HPV positive head and neck cancer.

## INTRODUCTION

Human papillomaviruses (HPVs) are double-stranded DNA viruses that readily infect keratinocytes causing a variety of diseases in humans ranging from warts to cancer [1]. HPV is the most common sexually transmitted infection in the United States, with an estimated 80 percent of sexually active adults acquiring an HPV infection in their lifetime [2]. Most HPV infections are self-limiting, but persistent infection with high-risk HPV (HR-HPV) types is causative in anogenital and head and neck cancers. Of these HR-HPV types, HPV type 16 (HPV16) is the most prevalent in tumors worldwide [3, 4]. HPV16 is causative in around 50% of all cervical cancers and 80-90% of HPV positive head and neck cancers [1, 5]. These HPV-related cancers of the oropharynx have reached epidemic proportions in the last decade [5]; in the United States, over 11,000 new cases of HPV-related oropharyngeal cancer are diagnosed per year [5].

During infection HR-HPV infect the basal cells of the epithelium, thought to be stem cells [6]. Following infection, host transcription factors bind to the control region of the virus and activate transcription from the viral genome [7] resulting in expression of viral proteins essential for the viral life cycle. The E7 protein binds to pRb and relieves the repression of E2F1 resulting in expression of cellular proteins required for progression of the cell cycle while E6 binds to and mediates degradation of p53 [8]; the combined action of these oncogenes promotes proliferation of the infected cell. The viral proteins E1 and E2 interact with host factors to promote the replication of the viral genome [9-16]. Upon initial viral infection the copy number per cell increases to between 20-50 copies representing the establishment phase of the viral life cycle. During the differentiation of the infected epithelium, the viral copy number is held to between 20-50 copies; the maintenance phase of the viral life cycle. In the upper layers of the infected epithelium the viral genome copy number is increased to around 1000 copies per cell; the amplification stage of the viral life cycle [17]. At this point the viral structural proteins L1 and L2 are expressed and the replicated viral genomes encapsulated to form viral particles that then egress from the upper layers of the infected epithelium.

In order to enhance our understanding of the HPV16 life cycle, it is important that this complicated process can be recapitulated *in vitro*. There are two HR-HPV cell lines that have been established from pre-malignant cervical lesions: W12 (containing HPV16) [18] and CIN612 (containing HPV31) [19], and these have been used extensively to study the life cycle of both viruses. An alternative approach in the study of the viral life cycle has been to immortalize primary epithelial cells by transfecting HR-HPV genomes into primary cells isolated from neonate foreskin due to their availability [20-22]. The resulting cell lines have been used to not only study the HPV life cycle in epithelium, but also to introduce mutant genomes and decipher which viral proteins and/or host interacting partners are essential for a complete viral life cycle in differentiating epithelium. These studies have also revealed differences between HR-HPV genomes in their requirements for executing the viral life cycle. For example, HPV16 requires an interaction between E2 and Brd4 for the viral life cycle whereas HPV31 does not [23, 24]. It seems likely that there will also be differences between the HR-HPV life cycles in tissues of different origins.

Given the ongoing epidemic of HPV16 positive oropharyngeal cancer, we established a model to enhance our understanding of the HPV16 life cycle in oral keratinocytes. Using TERT immortalized oral keratinocytes (NOKs) [25] we recapitulate the HPV16 life cycle in several clones and confirm classic markers of the HPV16 life cycle: activation of the DNA damage response [26], degradation of p53 in some clones [27], expression of the E1^E4 protein during differentiation [28] and expression of E2 with amplification of the viral genome in the upper layers of differentiating epithelium [29]. This system allows comparison of the gene expression of NOKs+HPV16 with the parental cell line (NOKs) using RNA-seq. The results demonstrate an extensive re-programing of host gene expression by HPV16 in oral keratinocytes, including genes involved in the intrinsic and innate immune response that have not previously been shown to be targeted by HR-HPV. We report validation of several of these genes and demonstrate that the anti-viral gene set which is regulated by un-phosphorylated ISGF3 (STAT1-STAT2-IRF9), U-ISGF3 [30, 31], is downregulated by HPV16 in this oral keratinocyte model. To emphasize the relevance of our system we also studied HPV16 immortalized human tonsil keratinocytes; these cells had identical properties to our NOKs+HPV16 clones with regards life cycle markers and down regulation of innate immune response genes. To further investigate the validity of our system, we used data from The Cancer Genome Atlas (TCGA) to demonstrate that the U-ISGF3 gene set is also downregulated in HPV positive head and neck cancers when compared with HPV negative cancers. The results demonstrate that we have generated a robust HPV16 life cycle model for the study of this virus in oral keratinocytes, and that this model is directly relevant to some aspects of the viral reprograming of host gene expression in HPV positive head and neck cancers.

## RESULTS

### A novel HPV16 positive oral keratinocyte model

**Figure 1:**
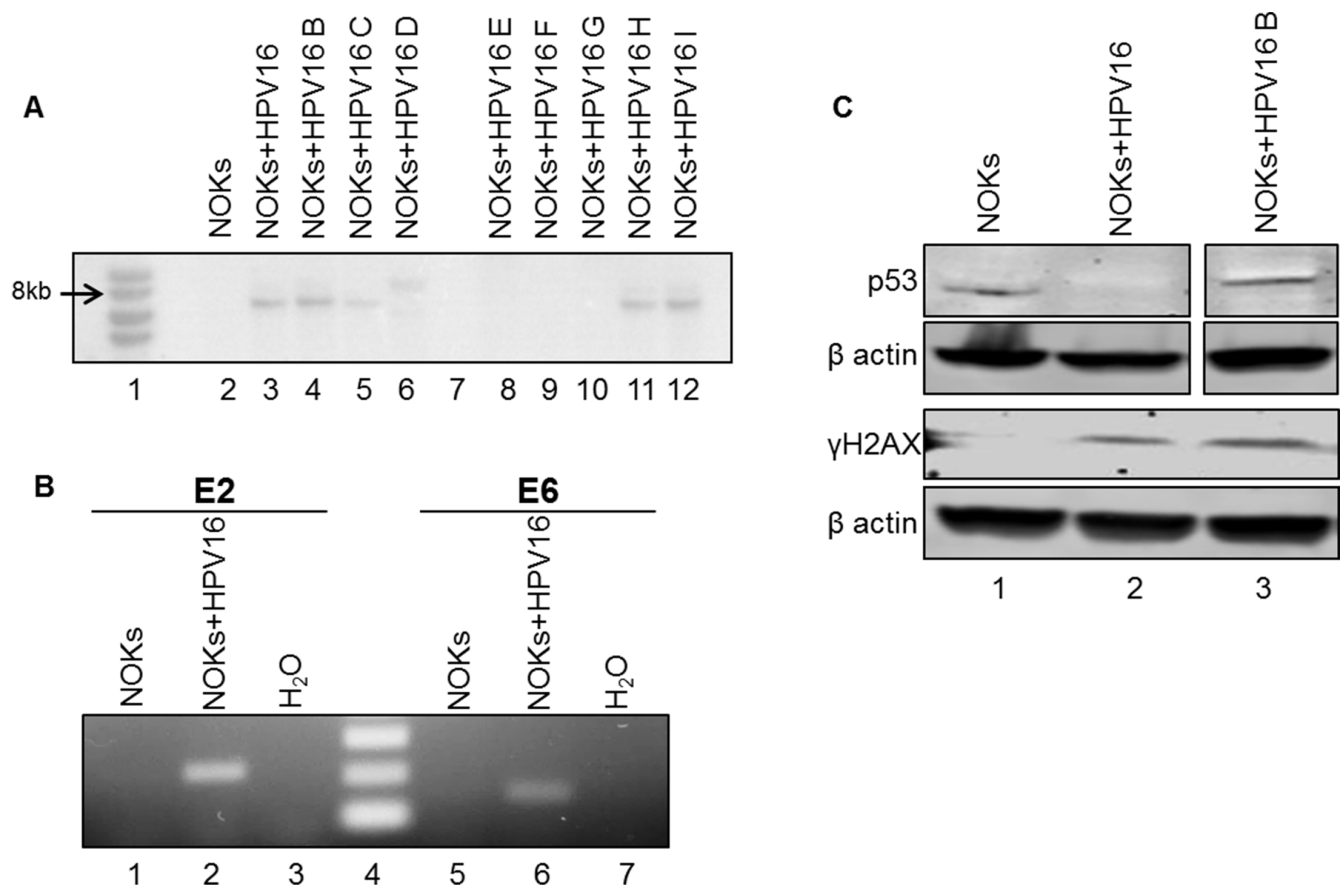
NOKs+HPV16 clones contain HPV16 episomes, express viral transcripts, and exhibits characteristic HPV-mediated cellular reprogramming **A.** Southern blot analysis using an HPV16 genome probe of NOKs+HPV16 clonal cell lines. **B.** RT-PCR analysis for E2 and E6 mRNA expression in NOKs and NOKs+HPV16. NOKs had neither gene expressed (1 and 4) while NOKs+HPV16 expressed both (2 and 5). Water control was negative (3 and 6). No band was detected in RNA samples not subjected to reverse transcriptase demonstrating that the bands are not amplified from contaminant viral genome DNA. **C.** Western blot analysis for p53, γH2AX , and β actin in NOKs, NOKs+HPV16 and NOKs+HPV16 B. p53 is downregulated in NOKs+HPV16 (compare lanes 1 and 2) but not in NOKs+HPV16 B while γH2AX is detected only in the presence of HPV16 demonstrating activation of the DNA damage response.

In order to study and enhance our understanding of the life cycle and pathogenesis of HPV16 in the oral cavity, a new model system was established utilizing TERT immortalized normal oral keratinocytes (NOKs). Immortalized NOKs cells were lipid transfected with a HPV16 containing vector alongside a vector expressing Cre recombinase [32]. The Cre recombinase targets Lox sites present in the plasmid to reconstitute the 8kbp HPV16 genome. Transfected cells were selected with G418 and expanded into clonal cell lines expressing the HPV16 genome. The decision to obtain a clonal cell line was based on the W12 and CIN612 cervical cell lines where clonal selection has identified cell lines maintaining episomal HPV16 [33] and HPV31 [34] genomes respectively. The parental W12 cells, for example, demonstrate instability for the retention of an episomal viral genome in long term cultures whereas clonal lines from this parent do retain stable episomes during extended culture [35]. Several NOKs+HPV16 clones were selected and expanded. DNA was harvested from the clones and digested with BamH1 (a single cutter of HPV16) and Dpn1 (to ensure any signal was not due to the input transfected DNA) and Southern blotting carried out to investigate the size of the HPV16 band detected (Figure 1a). Five clones, NOKs+HPV16, B, C, H, I, all contained bands of around 8kbp, the correct size for HPV16. No HPV16 bands were detected in the parental NOKs line (lane 1). To confirm that the viral genome is expressed in these cells, RNA was harvested from the clones and cDNA prepared; real-time PCR screening demonstrated the presence of E2 and E6 RNA in most clones, even some with very low levels of DNA (not shown). Figure 1b shows details of PCR carried out with primers targeting E2 and E6 on samples from NOKs (lanes 1 and 5) and NOKs+HPV16 (lanes 2 and 6) and also with H_2_O (lanes 3 and 7). Markers were loaded on lane 4. The expected bands of 216bp and 154bp were detected only in NOKs+HPV16 samples with the E2 and E6 primers respectively (lanes 2 and 6), demonstrating expression from the HPV16 genome. A similar protocol was carried out with the RNA in the absence of reverse transcriptase and no bands were detected demonstrating the signal is not generated from DNA contamination of the RNA (not shown).

HR-HPV activates the DNA damage response (DDR) in keratinocytes [26] and the E6 protein targets p53 for degradation [27]. We investigated the activation of the DDR and expression of p53 in NOKs+HPV16; Figure 1c. In the upper panel it is clear that there is reduced p53 expression in NOKs+HPV16 versus NOKs, but not in NOKs+HPV16 B; this discrepancy in p53 degradation by HPV16 is seen in other clonal systems and the reasons for this are not known. The lower panel demonstrates the presence of γH2AX (a marker for the DDR) in NOKs+HPV16 and NOKs+HPV16 B but not in NOKs.

The conclusions from Figure 1 is that NOKs+HPV16 contain an 8kbp HPV16 genome that is expressed at the RNA level, and that the presence of HPV16 has activated the DDR and degraded p53, two of the hallmarks of HR-HPV positive keratinocytes.

### NOKs+HPV16 can recapitulate the HPV16 life cycle

**Figure 2:**
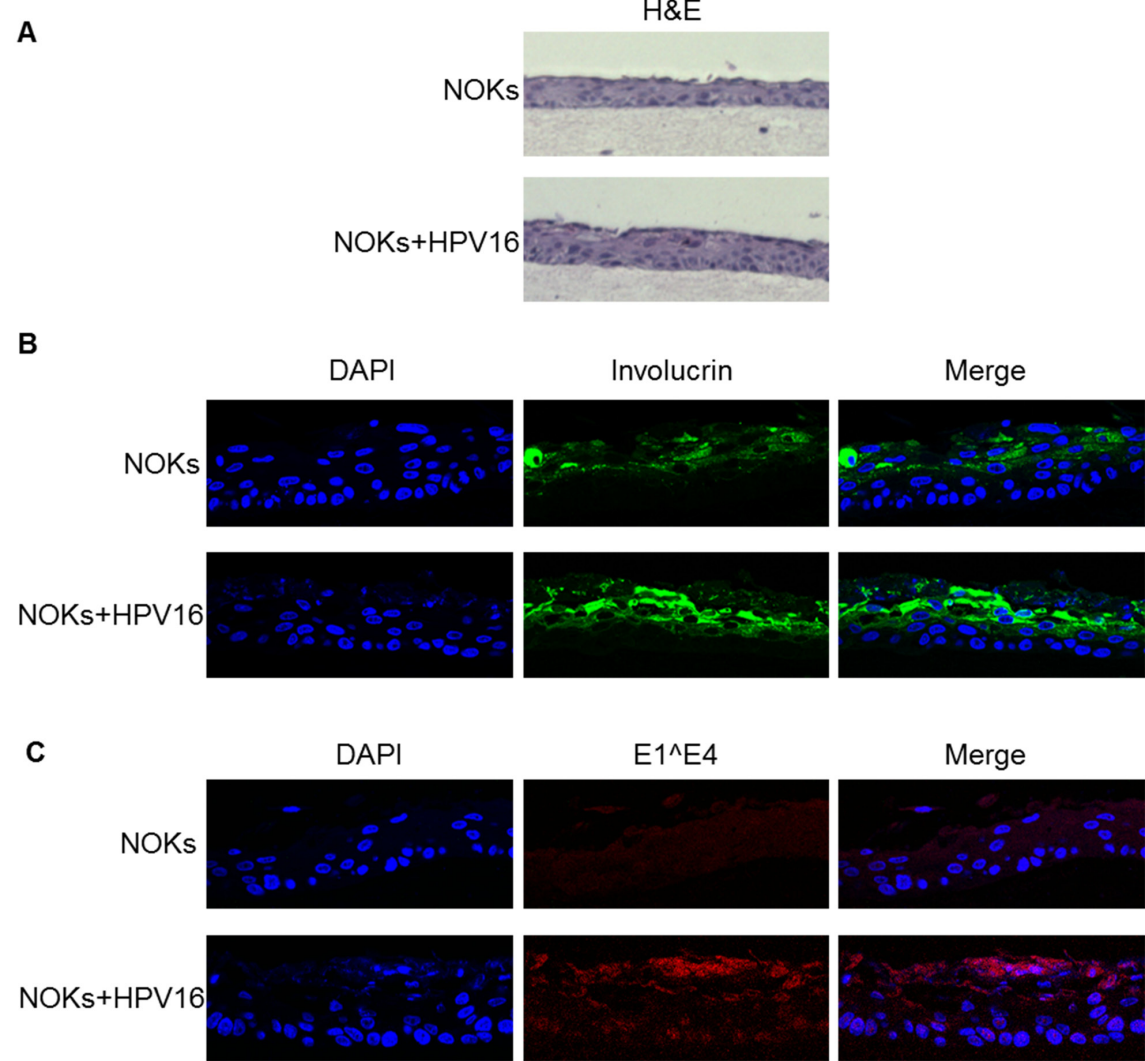
Organotypic raft staining of NOKs and NOKs+HPV16 Following rafting of NOKs and NOKs+HPV16 the skin equivalents were formalin fixed, paraffin embedded, sectioned and then stained. **A.** H&E staining demonstrates epithelial architecture in the NOKs and NOKs+HPV16. B) Involucrin staining demonstrates appropriate epithelial differentiation in NOKs and NOKs+HPV16. C) Protein expression of the E1^E4 HPV16 gene product is observed in NOKs+HPV16 only in the upper layers of the differentiating epithelium as expected.

**Figure 3:**
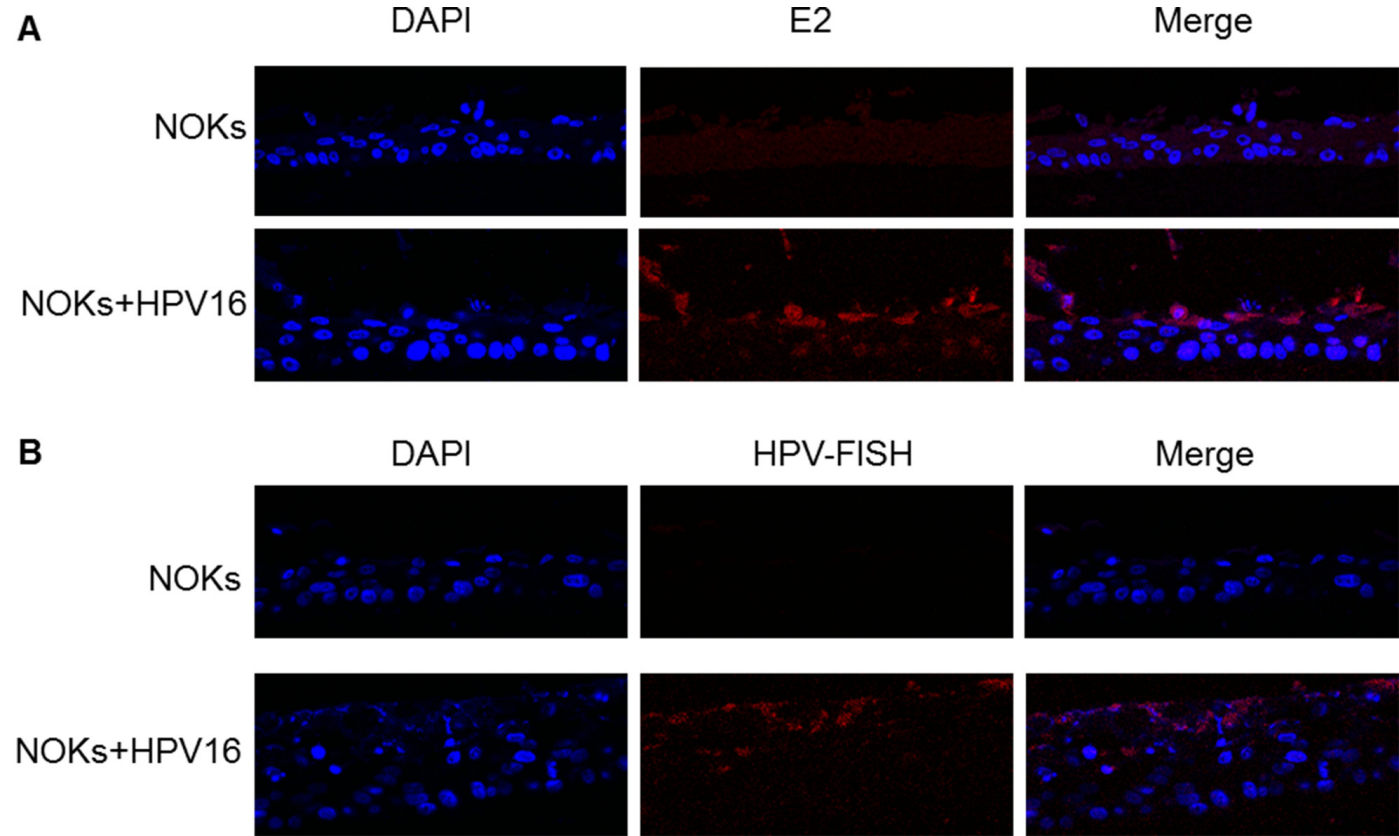
HPV16 replication factor expression and genome amplification in differentiated NOKs+HPV16 NOKs and NOKs+HPV16 organotypic raft cultures were stained with E2 antibody **A.,** and also with a fluorescent labeled HPV16 genome **B.** Detection of both E2 and the viral genome in the upper layers of the differentiated epithelium was only detected in NOKs+HPV16 (bottom panels in both figures) and not in NOKs (top panels).

To determine whether an HPV16 life cycle occurs in NOKs+HPV16 organotypic raft cultures were performed. The rafted cells were fixed, sectioned and stained to investigate the HPV16 life cycle. Figure 2a shows an H&E staining for NOKs and NOKs+HPV16. To confirm the correct differentiation of these cells, sections were stained with the differentiation marker involucrin, Figure 2b. Both NOKs and NOKs+HPV16 express involucrin in the mid and upperlayers of the differentiated epithelium, as would be expected; this demonstrates that the presence of HPV16 is not inhibiting the differentiation of these cells. To investigate whether the viral life cycle is occurring in the NOKs+HPV16 staining with an E1^E4 antibody was carried out, Figure 2c. A clear signal for this protein was detected only in NOKs+HPV16 revealing expression and splicing of E1^E4 from the HPV16 genome as would be expected during the HPV16 life cycle. To further confirm that NOKs+HPV16 support a viral life cycle, sections were investigated for the presence of the E2 protein and amplification of the viral genome. Figure 3a demonstrates that E2 is detected throughout the raft with an increase in the upper layers of NOKs+HPV16. Furthermore, FISH with the HPV16 genome detects amplification of the viral genome in the upper layers of the differentiated epithelium containing HPV16 only, Figure 3b. Both E2 and the HPV16 genome are detected, not only in the nucleus, but also in the cytoplasm of some cells in the upper layers of the raft indicating nuclear breakdown.

The conclusions from Figure 1-3 are that NOKs+HPV16 contain an episomal HPV16 genome which is capable of going through a life cycle in differentiating epithelium. The clonal nature of NOKs+HPV16 has given stability to the episomal nature of the viral genome. To confirm that this is not only observed in the NOKs+HPV16 clone, the staining for the viral proteins and genome amplification was repeated in NOKs+HPV16 B and the results are shown in Figure S1 demonstrating a successful life cycle.

### Host gene regulation by HPV16 in NOKs

The model system described in Figure 1-3 presents an opportunity to enhance our understanding of how HPV16 regulates the host genome in oral keratinocytes. NOKs and NOKs+HPV16 RNA samples were subjected to RNA-seq analysis as described in the Materials and Methods. Duplicate sample data were combined to demonstrate differential gene expression analysis revealing that 2624 genes were significantly differentially expressed 1.5 fold and greater in NOKs+HPV16 compared to NOKs. A full list of these genes is given in Supplementary Table S1. A list of the expression level of the HPV16 genes in the duplicates used to generate the gene expression data is given in Supplementary Table S2. Ingenuity Pathway Analysis (IPA) identified the top canonical pathways, upstream regulators, diseases and functions predicted to be altered in this data set; a summary of these is given in Supplementary Table S3. Many of the identified genes have not previously been shown to be regulated by any HPV protein in any system. Therefore, our oral keratinocyte model has allowed us to identify novel features of HPV16 host genome regulation in oral keratinocytes. The strength of our system is the clonal nature of NOKs+HPV16 combined with the availability of the parental NOKs cells for direct comparison. When immortalizing primary cells with HPV16, particularly tonsil cells, the parental cells have a limited life span making future studies and validation of gene expression changes difficult over the long term. Table S1 also shows the count number for reads from the RNA-seq data containing the HPV16 genes and as expected, all early genes are expressed. IPA analysis identified interferon signaling as one of the canonical pathways altered by HPV16 (predicted to be downregulated) and additionally a host of other innate and intrinsic immune response genes predicted to be downregulated. For example, HLA-A,B,C,F,H,K were all downregulated which would disrupt antigen presentation in the HPV16 containing cells.

### Recapitulating the HPV16 life cycle in primary human tonsil keratinocytes

**Figure 4:**
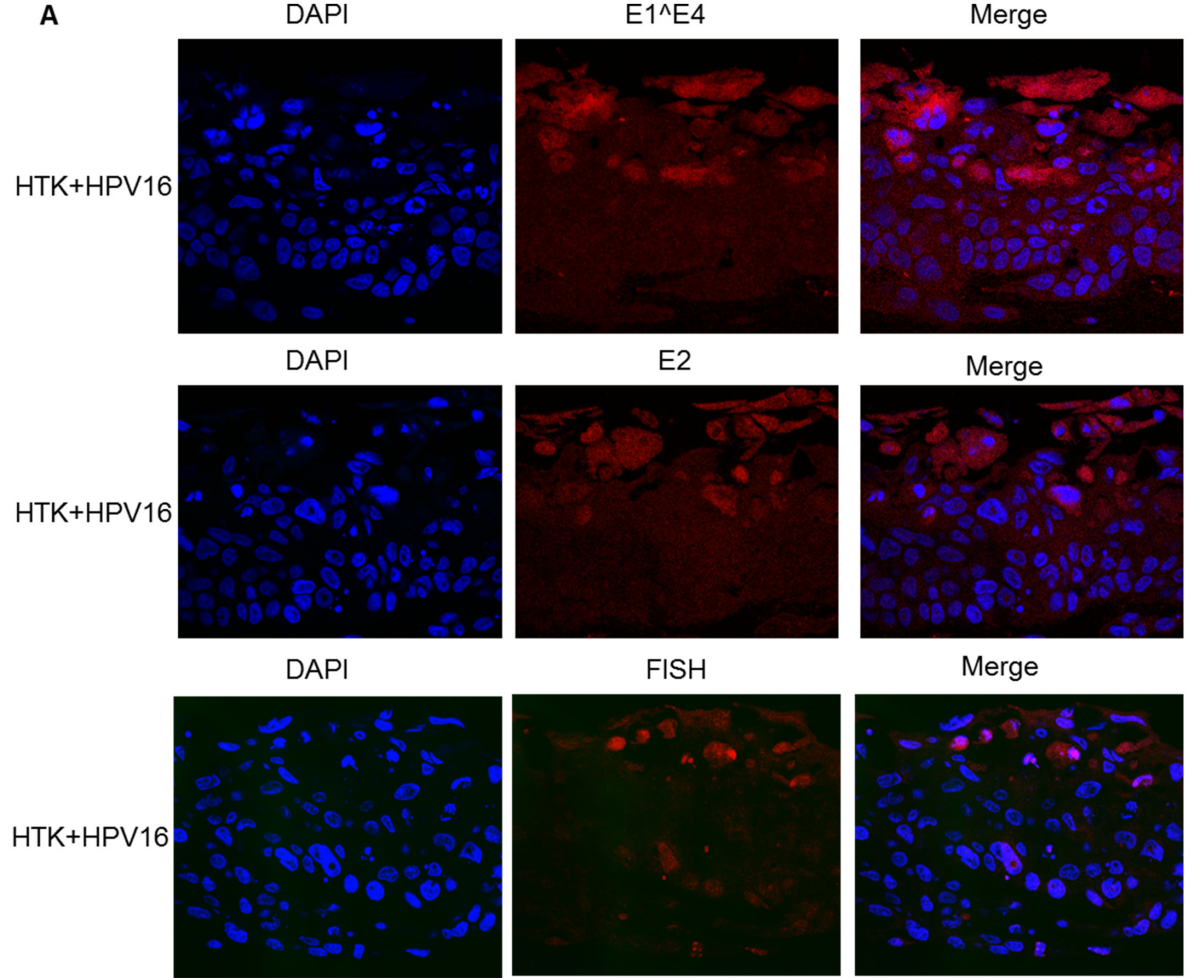
The HPV16 life cycle in HTK+HPV16 Primary human tonsil keratinocytes were immortalized with HPV16. They were then rafted, formalin fixed, paraffin embedded and stained with E1^E4 (A) and E2 (B) antibodies. Amplification of the viral genome in the upper layers of the epithelium was confirmed using FISH (C).

To enhance the relevance of our novel cell system, we also immortalized primary human tonsil keratinocytes with HPV16 as described [36]; HTK+HPV16. This cell line was then used to compare with our results generated in NOKs+HPV16 in subsequent studies. HTK+HPV16 contained an 8kbp sized viral genome on Southern blots following BamH1 digestion of DNA indicating an episomal viral genome (not shown). To confirm that the viral genome is episomal we recapitulated the life cycle of HPV16 using organotypic raft cultures followed by fixing and staining for viral proteins E1^E4 and E2 and amplification of the viral genome using FISH. The results are shown in Figure 4 and demonstrate the expression of the E1^E4 and E2 proteins in upper epithelial layers along with amplification of the HPV16 genome. This is identical to the results obtained with NOKs+HPV16 (Figures 2 and 3).

Others have observed down regulation of innate immune genes by HPV16 but our system has identified many novel targets, including IRF9, a member of the ISGF3 (interferon stimulataed gene factor 3) complex. We investigated the regulation of ISGF3 components and target genes in more depth in our oral keratinocyte model.

### Downregulation of the U-ISGF3 signature gene set by HPV16

**Figure 5:**
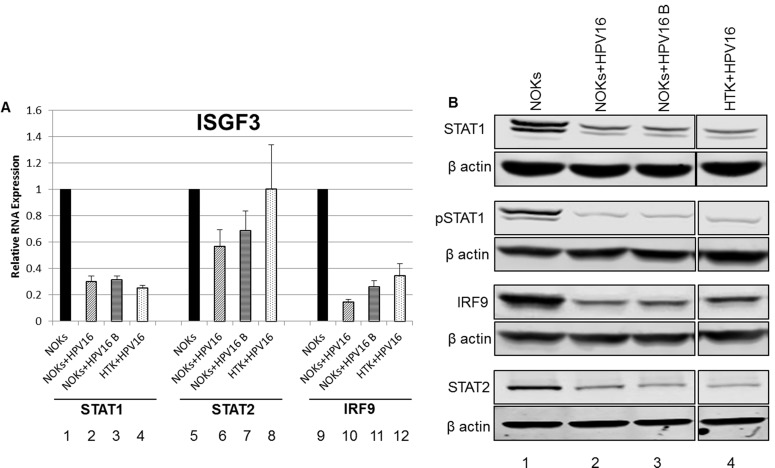
Reduced expression of ISGF3 components STAT1 and IRF9 in NOKs+HPV16 **A.** qRT-PCR analysis of STAT1, STAT2, and IRF9 mRNA expression levels in NOKs (1,5,9), NOKs+HPV16 (2,6,10), NOKs+HPV16 B (3,7,11) and HTK+HPV16 using GAPDH as an endogenous control gene. Data represents the average of 3 independent experiments and error bars indicate standard error of the mean. **B.** Western blot analysis for STAT1, pSTAT1, STAT2, IRF9, and β actin expression in NOKs (1), NOKs+HPV16 (2), NOKs+HPV16 B (3) and HTK+HPV16 (4). Please note all lanes come from the same western blot, lanes were removed for clarity and this is shown as a clear gap to the HTK+HPV16 lane.

Following treatment with interferon, a signaling cascade promotes phosphorylation of STAT1 and STAT2 which form a ternary complex with IRF9 resulting in the transcriptional activator ISGF3 that locates to the nucleus and activates transcription of interferon stimulated genes (ISGs) [37]. ISGs include STAT1 and IRF9 and the increased expression of these factors extends several days following interferon treatment resulting in an un-phosphorylated (U)-ISGF3 complex that elevates expression of a 29 gene signature set. This signature set contains several genes whose products are anti-viral factors [30, 31]. Both STAT1 and IRF9 were predicted to be downregulated by HPV16 in the oral keratinocytes (Table S1); STAT1 is down regulated by HPV31 in cervical cells [38], however this is the first report of any HR-HPV down regulating IRF9 to our knowledge. Figure 5a demonstrates that the RNA for both STAT1 and IRF9 is downregulated in NOKs+HPV16 (lanes 2,6,10). NOKs+HPV16 B (lanes 3,7,11) and HTK+HPV16 (lanes 4,8,12) when compared with NOKs (lanes 1,5,9) while changes in STAT2 expression are much smaller. This repression continues at the protein level as shown in western blots (Figure 5b); both STAT1 and IRF9 protein expression is severely attenuated by HPV16. In addition, there is a large reduction in phosphorylated STAT1 levels (bottom left panel) that may be related to the overall reduction in the expression of STAT1 protein. Interestingly, even though the RNA levels of STAT2 are not dramatically decreased there is clearly a reduction of the STAT2 protein level in all HPV16 containing cells. This is the first demonstration of STAT2 and IRF9 targeting by HPV16 and the results demonstrate a coordinated down regulation of the ISGF3 complex by HPV16.

**Figure 6:**
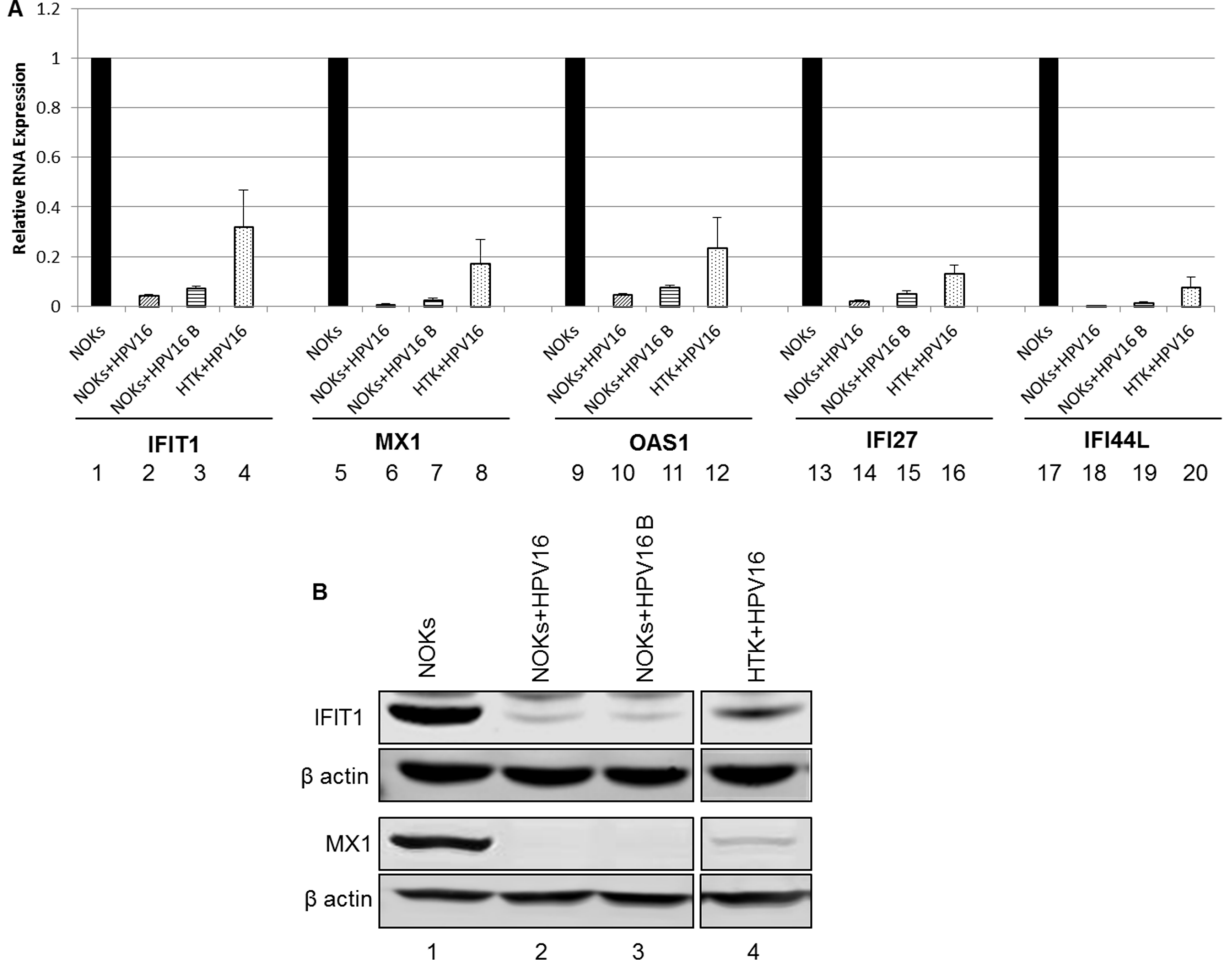
Reduced expression of innate immune response genes in HPV16 oral keratinocytes **A.** qRT-PCR analysis of mRNA expression levels of selected subset of U-ISGF3 controlled genes in NOKs (1,5,9,13,17), NOKs+HPV16 (2,6,10,14,18), NOKs+HPV16 B (3,7,11,15,19) and HTK+HPV16 (4,8,12,16,20) using GAPDH as an endogenous control gene. Data represents the average of 3 independent experiments and error bars indicate standard error of the mean. **B.** Western blot analysis for IFIT1, MX1, and β actin expression in NOKs (1), NOKs+HPV16 (2), NOKs+HPV16 B (3) and HTK+HPV16 (4). Please note all lanes come from the same western blot, lanes were removed for clarity and this is shown as a clear gap to the HTK+HPV16 lane.

Figure 4 confirms that unstimulated background levels of STAT1 and IRF9 are downregulated by HPV16. This is predicted to attenuate the function of U-ISGF3 in the HPV16 containing cells, and therefore expression of target genes of the U-ISGF3 complex was investigated. There are 29 genes that are direct targets of this complex [30, 31], many of them anti-viral genes, and they are listed in Table 1 with their corresponding expression level detected by RNA-seq comparing NOKs+HPV16 with NOKs shown. Of the 29 genes, 26 genes were predicted to be significantly differentially expressed at a magnitude >1.5 fold change and FDR <0.05. Strikingly, all 26 significantly differentially expressed genes were downregulated by HPV16. The three genes predicted not to be significantly down regulated, RARRES3, RTP4 and TMEM140, were all very poorly expressed in NOKs (not shown). To assess whether this observation was of significance, a chi-square goodness of fit test was performed which yielded a p-value of 3.64x10^-8^. Five of these genes (IFIT1, MX1, OAS1, IFI27, IFI44L) were chosen to validate this downregulation and the results from this experiment are shown in Figure 6a; all 5 of the RNA-seq downregulated genes showed reduced expression levels in NOKs+HPV16 (lanes 2,6,10,14,18), NOKs+HPV16 B (3,7,11,15,19) and HTK+HPV16 (4,8,12,16,20) when compared with NOKs (lanes 1,3,5,7,9,11). Two of these genes were chosen for validation at the protein level; IFIT1 and MX1. IFIT1 binds to and represses the replication properties of HPV18 E1 and may act similarly to block the function of HPV16 E1, making downregulation of this protein essential for promotion of the viral life cycle [39, 40]. MX1 was also investigated, a cellular protein that is known to target the envelope of certain viruses to block their infection. The results of these experiments are shown in Figure 5b; both IFIT1 and MX1 are downregulated at the protein level by HPV16 in all cells tested, reflecting their repression at the RNA level. Overall these results demonstrate that HPV16 directly targets the U-ISGF3 complex resulting in the downregulation of a set of anti-viral genes. The downregulation of this gene set is likely important in allowing the establishment and persistence of a HPV16 infection.

**Table 1:** The 29 genes listed in the left column are targets for U-ISGF3 (an un-phosphorylated complex of STAT1-STAT2-IRF9), the middle column lists the relative NOKs+HPV16/NOKs expression ratio for each gene and the right column lists FDR (genes with FDR >0.05 are not significantly differentially regulated in NOKs+HPV16 compared to NOKs).

	RNA-seq	FDR
**BATF2**	0.425284	0.009851
**BST2**	0.305074	1.94E-06
**DDX58**	0.398798	3.23E-08
**DDX60**	0.283276	1.26E-11
**EPSTI1**	0.575272	0.008519
**HERC5**	0.366456	1.86E-07
**HERC6**	0.452976	4.80E-05
**IFI27**	0.005244	3.45E-184
**IFI35**	0.372754	5.06E-06
**IFI44**	0.129795	9.02E-22
**IFI44L**	0.034289	2.10E-57
**IFIH1**	0.51146	1.16E-05
**IFIT1**	0.243442	1.95E-14
**IFIT3**	0.299616	7.93E-13
**IFITM1**	0.093663	1.01E-41
**IRF7**	0.258169	3.30E-14
**ISG15**	0.147926	1.38E-31
**MX1**	0.03692	7.55E-68
**MX2**	0.03936	5.15E-44
**OAS1**	0.114288	1.63E-31
**OAS2**	0.187293	6.56E-26
**OAS3**	0.428489	3.92E-05
**OASL**	0.390813	5.66E-08
**PLSCR1**	0.564845	0.000305
**RARRES3**	0.585854	0.107632
**RTP4**	1.043094	0.801762
**STAT1**	0.483738	1.52E-06
**TMEM140**	0.75585	0.161926
**XAF1**	0.175839	1.09E-07

### IFNκ as a potential master regulator of U-ISGF3 expression

**Figure 7:**
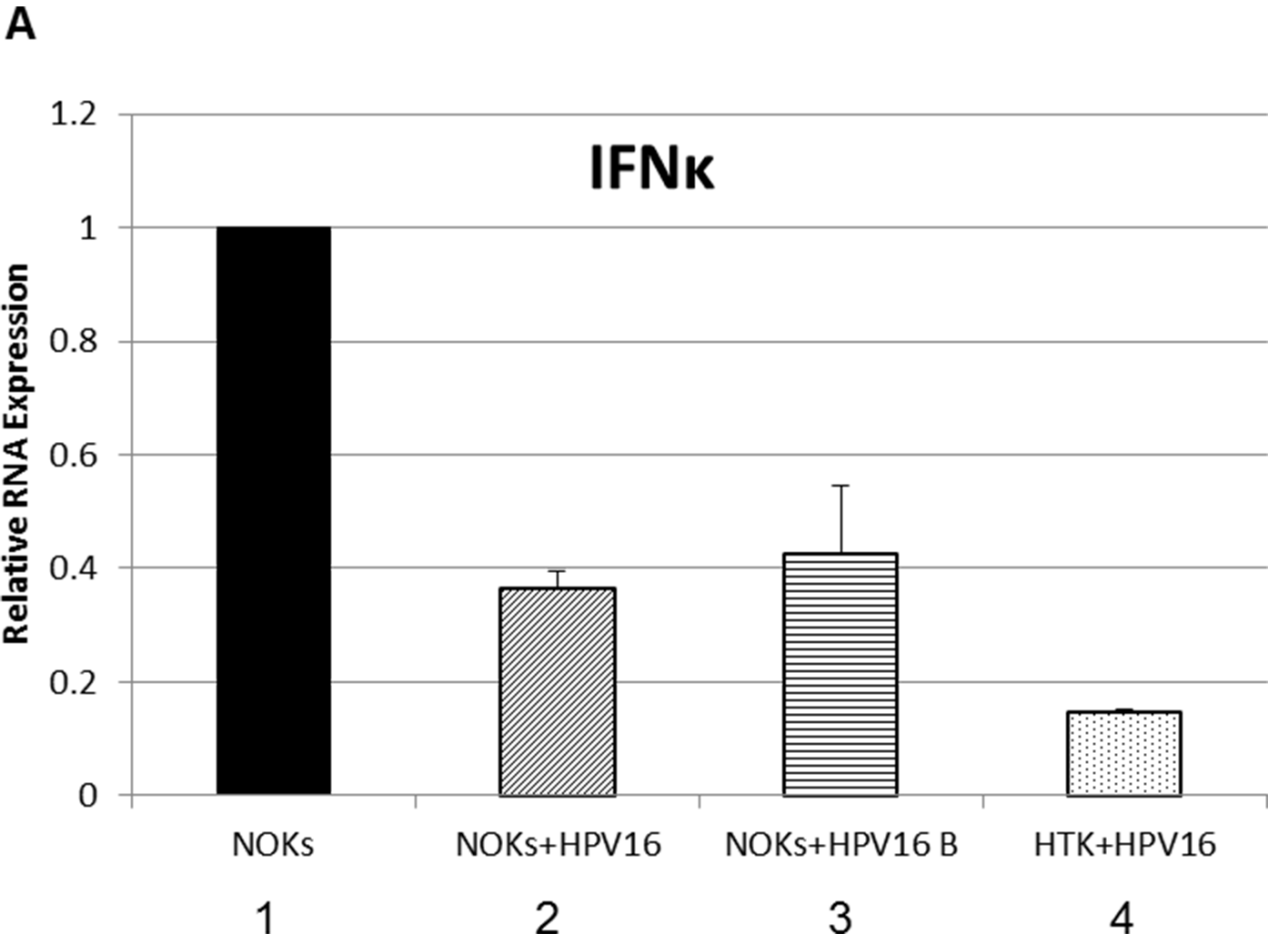
Interferon kappa expression is downregulated by HPV16 in HPV16 containing oral keratinocytes qRT-PCR analysis of mRNA expression levels of IFNβ in NOKs (1), NOKs+HPV16 (2), NOKs+HPV16 B (3) and HTK+HPV16 (4) using GAPDH as an endogenous control gene. Data represents the average of 3 independent experiments and error bars indicate standard error of the mean.

**Figure 8:**
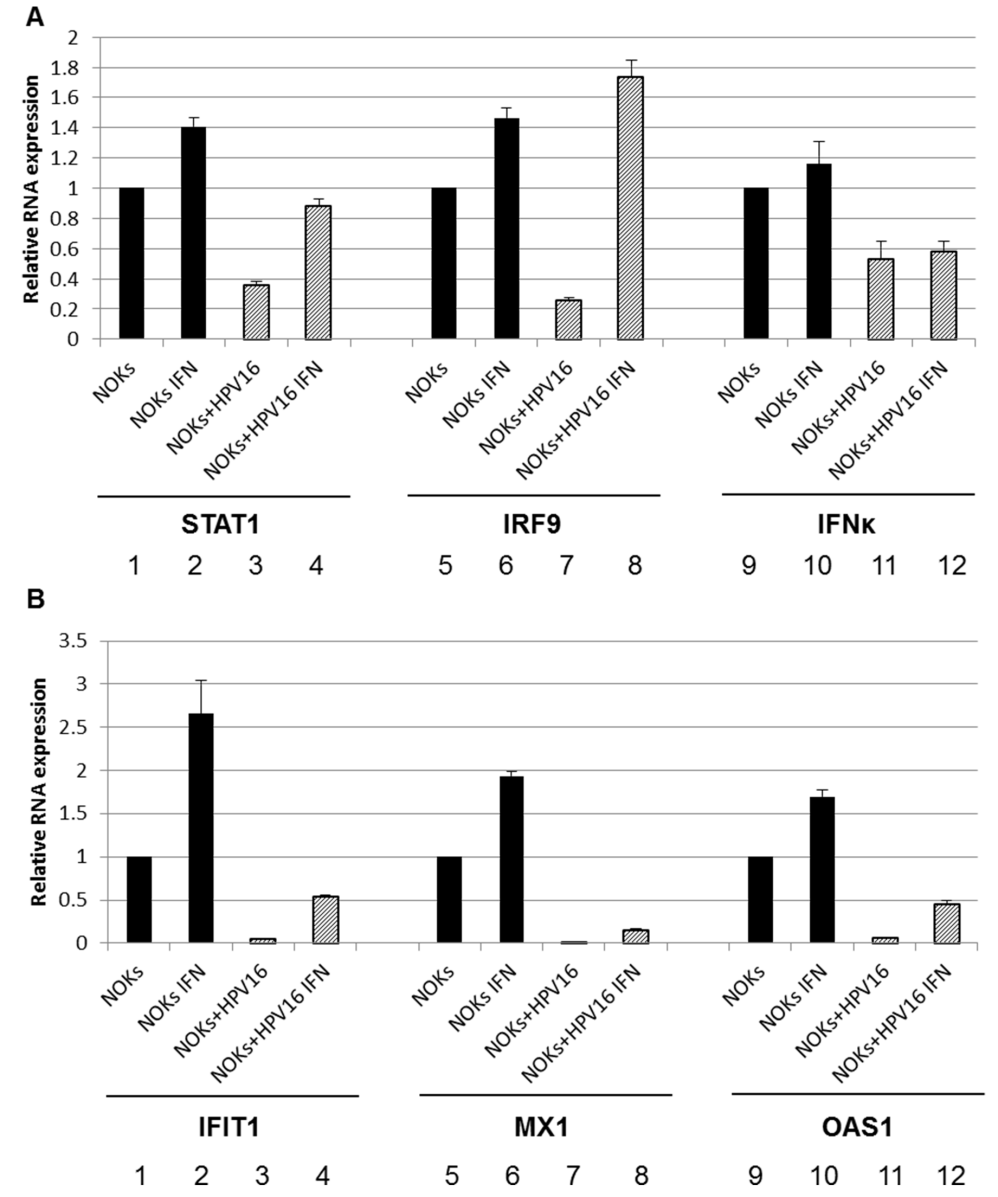
Inteferon β treatment restores expression of ISGF3 components but not targets in NOKs+HPV16 qRT-PCR analysis of mRNA expression levels of STAT1, IRF9, IFNk **A.**, and IFIT1, Mx1, OAS1 **B.** in NOKs (1,2,5,6,9,10) and NOKs+HPV16 (3,4,7,8,11,12) before (1,3,5,7,9,11) and after IFNβ (2,4,6,8,10,12) treatment. Results were standardized to GAPDH as an endogenous control and the data represents the average of 3 independent experiments and error bars indicated standard error of the mean.

Others have shown that IFNκ is the primary interferon expressed in keratinocytes and that attenuation of IFNκ is seen in both HPV-harboring foreskin keratinocytes and in HPV positive cervical cancer samples compared to non HPV- infected controls [41, 42]. IFNκ was also predicted to be repressed by HPV16 in our RNA-seq data set (Table S1). Therefore, the expression of IFNκ was specifically investigated. Figure 7 demonstrates that there is indeed a repression of IFNκ expression by HPV16 in NOKs+HPV16, NOKs+HPV16 B and HTK+HPV16. As interferon treatment results in the elevation of STAT1 and IRF9 gene expression, it seems likely that the reduction in expression of IFNκ contributes somewhat to the reduced expression of STAT1 and IRF9 and the consequent repression of the U-ISGF3 gene set by HPV16. Next, the expression of the STAT1 and IRF9 genes was investigated following interferon treatment of NOKs+HPV16 (Figure 8a). Both STAT1 and IRF9 RNA levels in NOKs+HPV16 were restored to similar levels observed in NOKs following treatment with IFNβ, another type 1 interferon (compare lane 4 with 2 and lane 8 with 6 respectively); IFNκ expression was not changed following IFNβ treatment (lanes 9-12). The expression of three of the U-ISGF3 genes, IFIT1, MX1 and OAS1, was then investigated following INFβ treatment of NOKs and NOKs+HPV16 and the results are shown in Figure 8b. The level of expression of these genes is not restored to the levels observed in NOKs following interferon treatment (compare lane 4 with 2; lane 8 with 6, and lane 12 with 10). These results suggest that HPV16 may have additional mechanisms that contribute towards the repression of the U-ISGF3 gene set, which are independent of IFNκ, STAT1 and IRF9 expression.

### The U-ISGF3 gene signature set is significantly downregulated in HPV positive *versus* HPV negative head and neck cancers

To further demonstrate the validity of our model system in the context of HPV-related head and neck cancers, RNA sequencing data from TCGA for 508 head and neck cancers was analyzed [43]. 60 of these head and neck cancer samples were HPV positive, while 448 of the samples were HPV negative. Focusing on U-ISGF3 controlled genes, our analysis revealed that 22 out of 29 U-ISGF3 controlled genes were significantly differentially expressed (FDR<0.05) in HPV positive samples and all were downregulated. Such concerted downregulation of this gene set is unlikely to occur by chance, and a chi-square goodness of fit yielded a significant p-value of 1.85x10^-5^. Of the 7 genes not predicted to be down regulated, 4 are not expressed well in NOKs; HERC5, RARRES3, RTP4 and TMEM140. The relative expression of U-ISGF3 genes in HPV positive samples compared to HPV negative samples is shown in Table 2. This downregulation of U-ISGF3 genes in HPV positive head and neck cancers overlaps significantly with the downregulation observed by RNA-seq in NOKs+HPV16 compared to NOKs. Overall, these results indicate that the downregulation of the innate immune response observed in NOKs+HPV16, particularly U-ISGF3 controlled genes, is representative of innate immune response downregulation in HPV positive head and neck cancers.

**Table 2:** The 29 genes listed in the left column are targets for U-ISGF3 (an un-phosphorylated complex of STAT1-STAT2-IRF9), column 2 lists the HPV+/HPV- head and neck cancer from The Cancer Genome Atlas expression ratio for each gene.

	TCGA	FDR	RNA-seq	FDR
**BATF2**	0.562668	0.000776	0.425284	0.009851
**BST2**	0.616193	0.000309	0.305074	1.94E-06
**DDX58**	0.62881	0.00015	0.398798	3.23E-08
**DDX60**	0.611391	0.000149	0.283276	1.26E-11
**EPSTI1**	0.603019	0.000166	0.575272	0.008519
**HERC5**	1.245769	0.067896	0.366456	1.86E-07
**HERC6**	0.643763	0.000774	0.452976	4.80E-05
**IFI27**	0.426227	4.63E-12	0.005244	3.45E-184
**IFI35**	0.659452	0.00016	0.372754	5.06E-06
**IFI44**	0.619862	0.000159	0.129795	9.02E-22
**IFI44L**	0.680138	0.012466	0.034289	2.10E-57
**IFIH1**	0.687302	0.000737	0.51146	1.16E-05
**IFIT1**	0.306364	1.08E-14	0.243442	1.95E-14
**IFIT3**	0.472905	4.11E-07	0.299616	7.93E-13
**IFITM1**	0.814065	0.07721	0.093663	1.01E-41
**IRF7**	0.758632	0.012251	0.258169	3.30E-14
**ISG15**	0.361944	2.12E-11	0.147926	1.38E-31
**MX1**	0.7503	0.026991	0.03692	7.55E-68
**MX2**	0.644413	0.000758	0.03936	5.15E-44
**OAS1**	0.743864	0.007154	0.114288	1.63E-31
**OAS2**	0.637834	0.000132	0.187293	6.56E-26
**OAS3**	0.714357	0.00245	0.428489	3.92E-05
**OASL**	0.547025	0.000145	0.390813	5.66E-08
**PLSCR1**	1.064071	0.462276	0.564845	0.000305
**RARRES3**	0.993157	0.959483	0.585854	0.107632
**RTP4**	0.908744	0.451218	1.043094	0.801762
**STAT1**	0.824422	0.067989	0.483738	1.52E-06
**TMEM140**	0.988175	0.894325	0.75585	0.161926
**XAF1**	0.629116	0.00013	0.175839	1.09E-07

Column 4 lists the NOKs+HPV16/NOKs expression ratio for each gene while Columns 3 and 5 lists FDR (genes with FDR >0.05 are not significantly differentially regulated) respectively.

## DISCUSSION

This paper describes a novel oral keratinocyte model for the study of the HPV16 life cycle. This is important, as there is an epidemic of HPV positive head and neck cancers (80-90% of which are caused by HPV16) [5] and novel diagnostics and treatments are desperately required to combat this disease. Therefore, enhancing our understanding of HPV16 in oral keratinocytes is a priority. The model presented in this report supports an HPV16 life cycle as demonstrated by E1^E4 and E2 expression in the differentiating epithelium as well as amplification of the viral genome as detected by FISH. This demonstrates the episomal nature of the viral genome present in NOKs+HPV16, NOKs+HPV16 B and HTK+HPV16. The clonal nature of NOKs+HPV16 has provided stability to the viral genome which has remained episomal over several months (not shown). This is similar to the W12 clone that was isolated that maintains an episomal HPV16 genome [33]. This clone was prepared from the original W12 cell line established from a cervical lesion [18]. Similarly, the CIN612-9E cell line is a clone from CIN612 cells and maintains the HPV31 genome as an episome [19].

While the generation of HPV16 virions from tonsil keratinocytes has been demonstrated [36], this is the first report, to our knowledge, that demonstrates in an oral keratinocyte model system E1^E4 and E2 expression in the differentiating epithelium along with viral genome amplification in the upper layers of the rafted tissue. A recent publication demonstrated that HPV18 can establish episomal genomes in NOKs [44]. Interestingly, this report demonstrated that there is no consistent HPV18 genome amplification in the upper layers of rafted NOKs containing the HPV18 genome, nor was their consistent expression of the HPV18 E1^E4 protein in the differentiated epithelium. HPV18 is very rarely detected in HPV positive head and neck cancer, unlike in cervical where it is detected in 20% of cases. Our data with HPV16 and those reported for HPV18 suggests there may be a tissue tropism for the HPV18 life cycle that would help explain the lack of HPV18 in HPV positive head and neck cancer.

RNA-seq analysis comparing gene expression in NOKs+HPV16 versus NOKs identified over 2,600 genes that were differentially expressed at a magnitude of 1.5 fold and higher. One of the striking features from the HPV16 downregulated genes we report here was a consistent down regulation of innate immune response genes, including many known interferon stimulated genes. Others have described sub-sets of these genes being targeted by HR-HPV in keratinocytes [38, 41, 45, 46]. However, the results presented here demonstrate several novel observations. The down regulation of IRF9 and STAT2 by HPV16 has not been reported before. Along with down regulation of STAT1 expression, an already identified HR-HPV target [38], the down regulation of IRF9 demonstrates that HPV16 directly targets the ISGF3 complex for down regulation in oral keratinocytes. ISGF3 is a transcription factor complex comprised of STAT1, IRF9 and STAT2 [37]. Interferon treatment stimulates phosphorylation of STAT1 and STAT2 in the cytoplasm promoting their dimerization and complexing with IRF9 resulting in an ISGF3 complex that translocates to the nucleus. Phosphorylated ISGF3 binds to specific target sequences in interferon stimulated genes and activates their transcription; therefore, the ISGF3 complex is an essential component of the innate immune response targeted by HPV16. Target genes of phosphorylated ISGF3 include STAT1 and IRF9, which themselves are regulated by a positive feedback loop. The induced STAT1 and IRF9 transcript and protein levels persist for several days following interferon treatment, even when the signaling cascade has dampened down. This results in a complex termed unphosphorylated ISGF3 (U-ISGF3) and a subset of 29 interferon stimulated genes have been identified as targets of U-ISGF3 [30, 31]. As STAT1 and IRF9 are suppressed by HPV16 it is perhaps unsurprising that 26 of the 29 U-ISGF3 genes are downregulated in NOKs+HPV16 versus NOKs. What is striking about this gene set is that most of them have assigned anti-viral activity [30]. For example, IFIT1 is known to complex with HPV18 E1 and block viral replication by retaining E1 in the cytoplasm [47, 48]. The conservation between HPV16 and 18 E1 is high and we predict that IFIT1 will also inhibit HPV16 E1-E2 replication via binding and retaining E1 in the cytoplasm.We are currently investigating this. Clearly, if IFIT1 is an inhibitor of HPV16 E1 function targeting downregulation of this gene and protein would be required for the viral life cycle and in particular viral genome amplification (Figure 4).

Stubenrauch and colleagues observed downregulation of a sub-set of the U-ISGF3 target set by HR-HPV and proposed that this was due to down regulation of IFNκ [41]. Others have also shown IFNκ targeting by HPV16 [42]. The data presented here supports a role for IFNκ in down regulation of U-ISGF3 as this gene is downregulated by HPV16 in NOKs. However, the results suggest that additional mechanism(s) are involved in the suppression of the U-ISGF3 target gene set. When NOKs and NOKs+HPV16 are treated with IFNβ (there is no commercial IFNκ; both interferons are proposed to operate via essentially the same receptors and signaling pathways) STAT1 and IRF9 levels increase to similar levels in NOKs and NOKs+HPV16. However, the expression of target genes such as IFIT1 and MX1 remain much lower in NOKs+HPV16 versus NOKs. This suggests that HPV16 has further mechanisms, in addition to STAT1 and IRF9 suppression, to target interferon signaling. For example, HPV16 E6 can bind to TYK2 and hamper phosphorylation of STAT1 and STAT2 [49]; and E7 complexes with cytosolic IRF9 preventing translocation to the nucleus therefore hindering ISGF3 function [50, 51]. However, the differences in ISGF3 target gene induction between NOKs and NOKs+HPV16 may be due to timing of cell harvest following interferon treatment; eventually levels of these genes could reach the same levels in both cell types, but there is at least a delay in this response in the HPV16 positive line. Our understanding of the overall mechanism that HPV16 uses to suppress interferon signaling remains incomplete.

The clonal nature of the NOKs+HPV16 with the corresponding NOKs parental line allowed a sensitive detection of host gene reprograming by HPV16. To investigate whether this regulation had relevance to *in vivo* HPV16 lesions, data from The Cancer Genome Atlas was exploited. Recently we reported on our analysis of the status of the viral genome in HPV16 positive tumors which demonstrates that E2 RNA is expressed in around three quarters of these tumors and in all likelihood the viral genome replicates as an episome in these E2 positive tumors, either by itself or in conjunction with fragments of host DNA [43]. Others agree with the high percentage of HPV positive head and neck cancers that retain expression of E2 RNA [52]. Head and neck cancer allows a comparison between HPV positive and negative tumors with regard gene expression as there are sets of both types of tumors in TCGA data; this cannot be done for cervical cancer as the large majority of the tumors are HR-HPV positive. Our analysis demonstrated that, of the 29 U-ISGF3 target genes, 22 were downregulated in HPV16 positive versus negative head and neck cancers and the chances of this being a random event are negligible. This is a similar number to that downregulated in NOKs+HPV16 versus NOKs. It should be noted that the levels of downregulation observed in the HPV16 positive versus negative TCGA data set are lower than that observed between NOKs+HPV16 versus NOKs. This is to be expected due to the differences in anatomical origin in the control HPV negative tumor set as data from all locations of the head and neck are included.

HPV positive head and neck cancer have, in general, a better response to radiation therapy than non-HPV tumors and while there is likely a role for p53 in this process it is unlikely to be the entire reason [53]. Another notable observation related to the downregulation of U-ISGF3 is that a number of the genes present in this gene set are also in the interferon related DNA damage resistance signature (IRDS). This gene set is *activated* in radiation resistance cancer cells [54, 55]. Therefore, suppression of members of the U-ISGF3 target genes that are in the IRDS gene set could also contribute to radiation sensitivity in HR-HPV positive tumors.

This report describes a new HPV16 life cycle model in oral keratinocytes. The current epidemic of HR-HPV positive head and neck cancers (80-90% of which are caused by HPV16) currently has no viral-based diagnostics or therapeutics for combating this disease. Therefore, it is a priority to enhance our understanding of HPV16 in oral keratinocytes. Using RNA-seq we determined extensive gene regulation by HPV16 in oral keratinocytes and we focused on validating this regulation by studying a set of genes targeted by the innate immune system. In addition, similar targeting of this innate immune gene set is observed in HPV16 positive head and neck cancers. Given the current promise of immunotherapy, understanding and reversing the repression of this set of innate immune response genes offers the opportunity to enhance the response of HPV positive tumors to therapy. We propose our new system as an ideal model to investigate the innate immune response, in addition to other pathways targeted by HPV16 in oral keratinocytes.

## MATERIALS AND METHODS

### Cell culture and generation of stable HPV16 full genome clones

Normal oral keratinocytes (NOKs) immortalized by TERT were supplied by Karl Munger (Tufts University) [25]. Stable NOKs clones expressing the full HPV16 genome were generated using a lipid transfection protocol utilizing the Cre/LoxP system as described previously [32]. Briefly, 1x10^6^ NOKs were seeded in 100mm^2^ plates. The next day NOKs were transfected using Lipofectamine 2000 (Invitrogen) with 1µg HPV16 vector alongside a pCDNA3 plasmid encoding pCre. Cells were monitored and media replaced every 3-4 days for 14 days after the initial G418 treatment. Individual colonies of surviving cells were then selected using cloning rings and transferred to 6 well plates containing G418 (Corning) supplemented keratinocyte serum-free medium (K-SFM) (Gibco). Candidate clones were then expanded in 100mm^2^ plates and the presence of HPV16 full genome was confirmed via RT-PCR for E2 and E6 RNA. Cell lines were passaged every 3-4 days and routinely checked for mycoplasma contamination.

NOKs and NOKs+HPV16 cells were grown in K-SFM with 1% (v/v) penicillin/streptomycin mixture (Life Technologies) containing 4 µg/mL hygromycin B (Sigma) at 37°C in a 5% CO_2_/95% air atmosphere and passaged every 3-4 days. NOKs+HPV16 were grown in the same medium also containing 150 µg/mL G418.

Human tonsil keratinocytes were immortalized using HPV16 derived from plasmid DNA using techniques described previously to generate HTK+HPV16 [36] and were a kind gift from Dr. Craig Meyers, Penn State.

Organotypic raft culture, Immunofluorescence staining and FISH NOKs, NOKs+HPV16 and HTK+HPV16 cells were differentiated via organotypic raft culture as described previously [56, 57]. Briefly, cells were seeded onto type 1 collagen matrices containing J2 3T3 fibroblast feeder cells. Cells were then grown to confluency atop the collagen matrices, which were then lifted onto wire grids. Wire grids were placed in cell culture dishes at the air-liquid interface so that raft cultures could be fed by diffusion of E-media. Raft cultures were allowed to stratify and differentiate for 13 days, with media replacement on alternate days.

Rafted samples were fixed with formaldehyde (4% v/v) and embedded in paraffin blocks. Multiple 4μm sections were cut from each sample. Sections were stained with hematoxylin and eosin (H&E) and others prepared for immunofluorescent staining as described previously [58]. Antibodies used and relevant dilutions are as follows: Involucrin (1/1000, Abcam), E1^E4 (1/50, Abcam), E2 (1/100, Abcam). Immune complexes were visualized using Alexa 488- or Alexa 595-labeled anti-species specific antibody conjugates (Molecular Probes). Cellular DNA was stained with 4’,6-diamidino-2-phenylindole (DAPI, Santa Cruz sc-3598). Microscopy was performed at the VCU Microscopy Facility, supported, in part, by funding from NIH-NCI cancer center grant P30 CA16059. Immunofluorescence was observed using a LSM 710 Laser Scanning Microscope and ZEN 2011 software (Carl Zeiss). Images were assembled in Adobe Photoshop 6.0.

Fluorescent in situ hybridization (FISH) staining for HPV16 genomes was performed as described previously on rafted samples [59].

### SYBR green qRT-PCR

*SYBR Green qRT-PCR*. 1x10^6^ cells were plated onto 100mm plates, trypsinized and pelleted after 24hrs and washed twice with phosphate buffered saline (PBS). RNA was immediately isolated using the SV Total RNA Isolation System (Promega) following the manufacturer’s instructions. Two micrograms of RNA were reverse transcribed into cDNA using the High Capacity Reverse Transcription Kit (Applied Biosystems). cDNA and relevant primers were added to PowerUp SYBR Green Master Mix (Applied Biosystems) and real-time PCR performed using 7500 Fast Real-Time PCR System (Applied Biosystems). Results shown are the average of three independent experiments with relative quantity of genes determined by the ΔΔCt method normalized to the endogenous control gene GAPDH.

*Primers:* GAPDH, IFIT1, MX1, OAS1, IFI27, IFI35, IFI44L primers used were designed by Qiagen (QuantiTech primer assay). Other primer pairs used in this study are as follows: STAT1 (Invitrogen): 5’-CAGCTTGACTCAAAATTCCTGGA-3’ (forward) and 5’-TGAAGATTACGCTTGCTTTTCCT-3’ (reverse). STAT2 (Invitrogen): 5’-CCAGCTTTACTCGCACAGC-3’ (forward) and 5’-AGCCTTGGAATCATCACTCCC-3’ (reverse). IRF9 (Invitrogen): 5’-GCCCTACAAGGTGTATCAGTTG-3’ (forward) and 5’-TGCTGTCGCTTTGATGGTACT-3’ (reverse). IFN-k (Invitrogen): 5’-GTGGCTTGAGATCCTTATGGGT-3’ (forward) and 5’-CAGATTTTGCCAGGTGACTCTT-3’ (reverse). HPV16 E2 (Invitrogen): 5’-tggaagtgcagtttgatgga-3’ (forward) and 5’-ccgcatgaacttcccatact-3’ (reverse). HPV16 E6 (Invitrogen): 5’-aatgtttcaggacccacagg- 3’ (forward) and 5’-gcataaatcccgaaaagcaa-3’ (reverse).

### Western blotting

Cells were trypsinized, washed twice with PBS, pelleted, and then resuspended in 50µl of lysis buffer (0.5% Nonidet P-40, 50mM Tris, ph 7.8, 150mM NaCl) supplemented with protease inhibitor (Roche Molecular Biochemicals) and phosphatase inhibitor cocktail (Sigma). The cell and lysis buffer mixture was incubated on ice for 30 min, centrifuged for 20 min at 184,000 rfc at 4°C, and supernatant was collected. Protein levels were determined utilizing the Bio-rad protein estimation assay (Bio-rad). Equal amounts of protein were boiled in 2x Laemmli sample buffer (Bio-rad). Samples were then loaded into a Novex 4-12% gradient Tris-glycine gel (Invitrogen), run at 100V for approximately 2 hours, and then transferred onto nitrocellulose membranes (Bio-rad) at 30V overnight using the wet blot method. Membranes were blocked in Odyssey blocking buffer (diluted 1:1 with PBS) at room temperature for 6 h and probed with relevant antibody diluted in Odyssey blocking buffer overnight at 4°C. Membranes were then washed with PBS supplemented with 0.1% Tween (PBS-Tween) before probing with corresponding Odyssey secondary antibody (goat anti-mouse IRdye800cw or goat anti-rabbit IRdye680cw) diluted 1:10,000 for 1h at 4°C. Membranes underwent washing in PBS-Tween before infrared scanning using the Odyssey CLx Li-Cor imaging system. The following antibodies were used for western blot analysis at 1:1000 dilutions in Odyssey blocking buffer (diluted 1:1 with PBS): IFIT1, MX1 (D3W7I), IRF9 (D8G7H) from Cell Signaling Technology. STAT1 (sc-346), STAT2 (sc-1668), pSTAT1 Tyr 701 (sc-135648), β-actin mouse (sc-81178), β-actin rabbit (sc-130656) from Santa Cruz Biotechnology. HPV16 E2 (TVG 261) from Abcam.

### Southern blotting

Total cellular DNA was extracted as described previously and analyzed by southern blotting using a (α-^32^P) dCTP-labeled HPV16 genomic probe [60]. DNA was digested with BamH1 to linearize the HPV16 episomal genomes and Dpn1 was included to insure that all input DNA was digested and not represented as replicating viral DNA.

### RNA sequencing

RNA was extracted using the same method described for SYBR Green qRT-PCR above. RNA sequencing was performed by the Genomics, Epigenomics and Sequencing core at the University of Cincinnati under the supervision of Dr. Xiang Zhang. Duplicates were sequenced for NOKs and NOKs+HPV16. The RNA concentration was measured by Nanodrop (Thermo Scientific) and its integrity was determined by Bioanalyzer (Agilent). The NEBNext Ultra Directional RNA Library Prep Kit (New England BioLabs) was used for library preparation, which used dUTP in cDNA synthesis to maintain strand specificity. In short, the isolated polyA RNA or rRNA/globin depleted RNA was Mg2+/heat fragmented (∼200 bp), reverse transcribed to 1st strand cDNA, followed by 2nd strand cDNA synthesis labelled with dUTP. The purified cDNA was end repaired and dA tailed, and then ligated to adapter with a stem-loop structure. The dUTP-labelled 2nd strand cDNA was removed by USER enzyme to maintain strand specificity. After indexing via PCR (∼12 cycles) enrichment, the amplified libraries together with library preparation negative control were cleaned up by AMPure XP beads for QC analysis. To check the quality and yield of the purified library, one µl library was analyzed by Bioanalyzer (Agilent) using DNA high sensitivity chip. To accurately quantify the library concentration for the clustering, the library was 1:104 diluted in dilution buffer (10 mM Tris-HCl, pH 8.0 with 0.05% Tween 20), and qPCR measured by Kapa Library Quantification kit (Kapabiosystem) using ABI’s 9700HT real-time PCR system (Thermo Fisher). Individually indexed and compatible libraries were proportionally pooled (∼25 million reads per sample) for clustering in cBot system (Illumina). Libraries at the final concentration of 15 pM were clustered onto a single read (SR) flow cell using Illumina TruSeq SR Cluster kit v3, and sequenced to 50 bp using TruSeq SBS kit on Illumina HiSeq system. To analyze differential gene expression he latest human assembly, Homo_sapiens.GRCH38.dna_sm.primary_assembly.fa was downloaded along with its corresponding .gtf file.

### Differential gene expression in NOKs

To analyze differential gene expression the latest human assembly, Homo_sapiens.GRCH38.dna_sm.primary_assembly.fa was downloaded from Ensembl along with its corresponding .gtf file. A reference file was created using bowtie2 build command. RNA-seq fastq files for NOKs and NOKs+HPV16 were locally aligned to GRCh38 reference sequence using bowtie2. Mapped reads were sorted by Samtools and BAM file were analyzed by DESeq2 [61]. Duplicates were collapsed and a pre-filter was used to eliminate any probe that did not have a count >1. Gene-level analysis was then performed as described previously and log2 fold change was converted to ratio of NOKs HPV+/NOKs HPV- [61]. Only gene expression differences >1.5 fold in magnitude between NOKs and NOKs+HPV16 were assessed and statistical significance was determined using a false discovery rate (FDR) of 0.05 with genes whose p value was less than the critical value considered to be statistically significant.

### Differential gene expression in TCGA HNSCC

RNA-seq Version 2 head and neck squamous cell carcinoma (HNSC) expression data was obtained from The Cancer Genome Atlas (TCGA) as described previously [43]. The RSEM expected counts for each patient/gene were rounded to the nearest whole number and matrix was analyzed by DESeq2 and gene level analysis was performed with the same method as stated above. To assess whether gene expression between comparison groups was considered statistically significant, a Benjamini and Hochberg correction was performed using a FDR of 0.05 and all genes whose p value was less than the critical value were considered to be statistically significant.

### Analysis of U-ISGF3 gene set

Chi square analysis was performed in order to determine whether the distribution of downregulated genes observed in the U-ISGF3 gene set was significantly different than the expected distribution of up and downregulated genes as modeled by the significant gene sets for RNA-seq or TCGA analysis described in the differential gene expression sections above.

## SUPPLEMENTARY MATERIALS FIGURES AND TABLES








