# Correction: An oral keratinocyte life cycle model identifies novel host genome regulation by human papillomavirus 16 relevant to HPV positive head and neck cancer

**DOI:** 10.18632/oncotarget.26962

**Published:** 2019-05-14

**Authors:** Michael R. Evans, Claire D. James, Oonagh Loughran, Tara J. Nulton, Xu Wang, Molly L. Bristol, Brad Windle, Iain M. Morgan

**Affiliations:** ^1^ Department of Oral and Craniofacial Molecular Biology, VCU Philips Institute for Oral Health Research, Virginia Commonwealth University School of Dentistry, Richmond, VA, USA; ^2^VCU Massey Cancer Center, Richmond, VA, USA

**This article has been corrected:** Due to errors in document preparation, The NOKs used in the original version of this manuscript were in fact N/Tert-1 (both are TERT immortalized, with NOKs being from gingival keratinocytes and N/Tert-1 from foreskin keratinocytes); the manuscript is now corrected accordingly. None of the results or the conclusions from the manuscript are in any way altered by this different genetic background; HPV16 reprograms N/Tert-1 cells similarly to HPV16 in head and neck cancer as determined by analysis of The Cancer Genome Atlas (TCGA). The HPV16 immortalized human tonsil keratinocytes remain a bridge between the results with HPV16 positive N/Tert-1 and TCGA data. The authors apologize for this error.

Original article: Oncotarget. 2017; 8:81892–81909 . https://doi.org/10.18632/oncotarget.18328

