# Supplementary material for: An oral keratinocyte life cycle model identifies novel host genome regulation by human papillomavirus 16 relevant to HPV positive head and neck cancer
Source: Oncotarget. 2017 Jun 1;8(47):81892–909. doi: 10.18632/oncotarget.18328 (PMC5669857; doi:10.18632/oncotarget.18328)
Supplement: Supplementary file 1 [file oncotarget-08-81892-s001.pdf]

An oral keratinocyte life cycle model identifies novel host genome regulation by human papillomavirus 16 relevant to HPV positive head and neck cancer

Supplementary Material

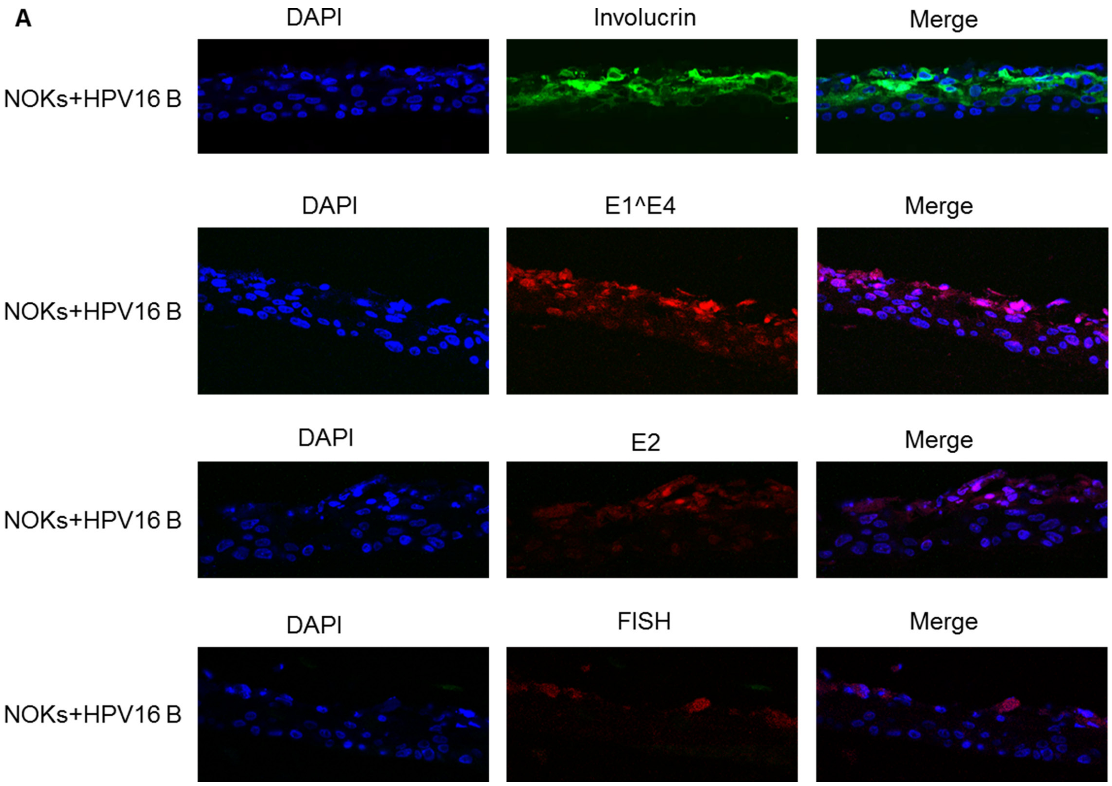

Supplementray Material: The HPV16 life cycle in NOKs+HPV16 B

For Supplementray Tables see in Supplementary Files
