# Supplementary material for: An oral keratinocyte life cycle model identifies novel host genome regulation by human papillomavirus 16 relevant to HPV positive head and neck cancer
Source: Oncotarget. 2017 Jun 1;8(47):81892–909. doi: 10.18632/oncotarget.18328 (PMC5669857; doi:10.18632/oncotarget.18328)
Supplement: Supplementary file 4 [file oncotarget-08-81892-s004.rtf]

Analysis Name: For IPA - 2017-02-02 10:27 AM Analysis Creation Date: 2017-02-02

Build version: 430520M

Content version: 31813283 (Release Date: 2016-12-05)


Analysis Settings

Reference set: Ingenuity Knowledge Base (Genes Only) Relationship to include: Direct and Indirect
Includes Endogenous Chemicals

Optional Analyses: My Pathways My List


Filter Summary:

Consider only relationships where confidence = Experimentally Observed


(c) 2000-2017 QIAGEN. All rights reserved.	1


Summary of Analysis - For IPA - 2017-02-02 10:27 AM

Top Canonical Pathways

Name	p-value	Overlap	
 Myc Mediated Apoptosis Signaling	3.57E-09	37.1 %	26/70	
 IGF-1 Signaling	5.61E-09	31.1 %	33/106	
 Pancreatic Adenocarcinoma Signaling	8.08E-09	29.7 %	35/118	
 Interferon Signaling	2.70E-08	47.2 %	17/36	
 Cell Cycle: G2/M DNA Damage Checkpoint Regulation	3.52E-08	40.8 %	20/49	


Top Upstream Regulators

Upstream Regulator	p-value of overlap	Predicted Activation	
 ESR1	1.52E-35	Activated	
 TP53	8.94E-32		
 MAPK1	4.39E-25	Activated	
 NUPR1	5.52E-24	Inhibited	
 dextran sulfate	5.59E-24		


Top Diseases and Bio Functions

Diseases and Disorders			
Name	p-value	#Molecules	
 Cancer	1.60E-06 - 1.00E-60	2071	
 Organismal Injury and Abnormalities	1.60E-06 - 1.00E-60	2081	
 Gastrointestinal Disease	1.08E-06 - 2.20E-58	1823	
 Hepatic System Disease	5.69E-07 - 5.98E-29	976	
 Reproductive System Disease	1.60E-06 - 2.34E-28	1124	


Molecular and Cellular Functions


(c) 2000-2017 QIAGEN. All rights reserved.	2		
			

Summary of Analysis - For IPA - 2017-02-02 10:27 AM

Name	p-value	#Molecules	
 Cellular Growth and Proliferation	1.00E-06 - 3.73E-35	914	
 Cell Death and Survival	1.61E-06 - 2.35E-33	774	
 Cell Cycle	1.35E-06 - 1.63E-32	448	
 Cellular Assembly and Organization	1.47E-06 - 3.18E-26	469	
 Cellular Function and Maintenance	1.40E-06 - 3.18E-26	640	


Physiological System Development and Function			
Name	p-value	#Molecules	
 Organismal Survival	8.95E-09 - 3.33E-23	580	
 Connective Tissue Development and Function	1.32E-06 - 1.16E-16	362	
 Tissue Development	1.62E-06 - 1.16E-16	717	
 Cardiovascular System Development and Function	9.65E-07 - 1.53E-16	345	
 Organismal Development	1.62E-06 - 1.24E-15	553	


Top Tox Functions

Assays: Clinical Chemistry and Hematology			
Name	p-value	#Molecules	
 Increased Levels of Red Blood Cells	5.75E-02 - 4.43E-03	20	
 Increased Levels of AST	6.49E-02 - 6.49E-02	4	
 Increased Levels of Hematocrit	7.13E-02 - 7.13E-02	15	
 Increased Levels of Potassium	9.57E-02 - 9.57E-02	4	
 Decreased Levels of Albumin	4.26E-01 - 1.05E-01	4	


Cardiotoxicity


(c) 2000-2017 QIAGEN. All rights reserved.	3		
			

Summary of Analysis - For IPA - 2017-02-02 10:27 AM

Name	p-value	#Molecules	
 Cardiac Necrosis/Cell Death	3.59E-01 - 8.57E-05	52	
 Cardiac Hypertrophy	2.84E-01 - 1.38E-04	71	
 Cardiac Output	3.25E-01 - 1.52E-03	12	
 Cardiac Arrythmia	1.00E00 - 2.39E-03	44	
 Congenital Heart Anomaly	6.32E-01 - 2.89E-03	43	


Hepatotoxicity			
Name	p-value	#Molecules	
 Liver Hyperplasia/Hyperproliferation	1.00E00 - 2.95E-27	924	
 Hepatocellular Carcinoma	1.00E00 - 1.24E-11	162	
 Liver Necrosis/Cell Death	5.79E-01 - 3.21E-07	60	
 Liver Proliferation	2.84E-01 - 5.71E-06	48	
 Liver Fibrosis	2.03E-01 - 4.73E-04	42	


Nephrotoxicity			
Name	p-value	#Molecules	
 Renal Proliferation	5.41E-01 - 4.05E-05	58	
 Renal Necrosis/Cell Death	5.89E-01 - 4.17E-05	89	
 Renal Damage	3.88E-01 - 1.81E-04	37	
 Renal Tubule Injury	3.88E-01 - 1.81E-04	24	
 Glomerular Injury	5.41E-01 - 3.76E-04	60	


Top Regulator Effect Networks

ID Regulators	Diseases & Functions	Consistency Score		
 1	ATM,miR-34a-5p (and other miRNAs w/seed	cell movement of endothelial cells (+7 more)	19.218		
	GGCAGUG) (+5 more)				

(c) 2000-2017 QIAGEN. All rights reserved.	4		
			

Summary of Analysis - For IPA - 2017-02-02 10:27 AM

 2	NPPB,RLIM	apoptosis,cell movement of endothelial cells (+5 more)	17.963		
 3	HOTAIR,IFNA10,IFNA14,IFNA16,IFNA21,IFNA4,IFNA5	antiviral response,replication of RNA virus (+1 more)	16.971		
	(+12 more)				
 4	ABCB4,FN1,GMNN,let-7 (+8 more)	apoptosis of myeloma cell lines (+7 more)	16.347		
 5	EFNA2,FOSB,JUNB,mir-1 (+5 more)	migration of tumor cell lines (+1 more)	13.377		


Top Networks

ID	Associated Network Functions	Score	
 1	DNA Replication, Recombination, and Repair, Cell Cycle, Cell Morphology	39	
 2	Cell Cycle, Cellular Assembly and Organization, DNA Replication, Recombination, and Repair	36	
 3	Cell Morphology, Cellular Assembly and Organization, Cellular Function and Maintenance	36	
 4	Post-Translational Modification, Cardiovascular Disease, Cell-To-Cell Signaling and Interaction	36	
 5	Cell Cycle, Cell-To-Cell Signaling and Interaction, Cellular Growth and Proliferation	36	


Top Tox Lists

Name	p-value	Overlap	
 Cell Cycle: G2/M DNA Damage Checkpoint Regulation	1.15E-07	38.5 %	20/52	
 Liver Necrosis/Cell Death	1.59E-07	20.7 %	61/294	
 p53 Signaling	3.18E-07	27.7 %	31/112	
 Liver Proliferation	2.69E-06	20.9 %	48/230	
 Renal Necrosis/Cell Death	5.22E-06	16.8 %	89/529	


Top Analysis-Ready Molecules

Expr Log Ratio up-regulated			
Molecules	Expr. Value	Expr. Chart	
 MYADM	6.194		
 PDPN	6.179		
 NECAB1	4.632		

(c) 2000-2017 QIAGEN. All rights reserved.	5		
			

Summary of Analysis - For IPA - 2017-02-02 10:27 AM

 C8orf88	4.243	
 SULT1E1	3.970	
 TNRC6C	3.775	
 ZNF608	3.765	
 IGFBP3	3.699	
 DENND2D	3.566	
 LMCD1	3.501	


Expr Log Ratio down-regulated			
Molecules	Expr. Value	Expr. Chart	
 IFI27	-7.575		
 FAM155B	-5.006		
 LCP1	-4.993		
 IFI44L	-4.866		
 MX1	-4.759		
 IFI6	-4.687		
 MX2	-4.667		
 DPYSL4	-4.195		
 DIP2C	-4.150		
 CYB5A	-3.931		


(c) 2000-2017 QIAGEN. All rights reserved.	6		
			
